# The effectiveness of lifestyle interventions to reduce side effects of androgen deprivation therapy for men with prostate cancer: a systematic review

**DOI:** 10.1007/s11136-019-02361-z

**Published:** 2019-12-12

**Authors:** Maud J. M. Geerkens, Nieck S. A. Pouwels, Harry P. Beerlage

**Affiliations:** Urology Department, Amsterdam UMC locatie AMC Netherlands, Meibergdreef 9, 1105 AZ Amsterdam, The Netherlands

**Keywords:** Males, ADT, Side effects, Prostate cancer, Lifestyle interventions

## Abstract

**Purpose:**

The aim of this review is to systematically review randomized controlled trials on lifestyle interventions on PCa patients undergoing androgen deprivation therapy.

**Methods:**

A literature search was conducted using the electronic databases Medline and PubMed. To be eligible, studies had to be randomized controlled trials (RCTs) that focused on side effects of ADT and lifestyle interventions to reduce side effects for men undergoing ADT with PCa. Lifestyle interventions were defined as interventions that included any dietary or behavioral components.

**Results:**

Twenty-nine trials were included. Most of them focused on exercise interventions, while some investigated the effect of dietary or behavioral interventions. The effect of different lifestyle influencing modalities aimed to improve on the adverse effects of ADT varied greatly.

**Conclusions:**

It is not possible to draw one conclusion on the effect of exercise-based interventions, but noted on several adverse effects of ADT improvement. Further studies are necessary to develop personalized lifestyle interventions in order to mitigate the adverse effects.

## Introduction

Prostate cancer is one of the most common forms of cancer in men and the second cause of death [1]. Androgens and androgen receptor signaling play an important role in the normal growth and function of the prostate, but also in the development and maintenance of prostate cancer. The beneficial effect of castration in the treatment of prostate cancer has already been discovered in 1941, by Huggins [2]. LHRH agonists are the most chosen form of chemical castration [3]. LHRH agonists bring the serum testosterone to a level similar to castration by interfering in the pulsatile release of LHRH in the hypothalamus, thereby down-regulating the release of luteinizing hormone in the anterior pituitary gland. Androgen deprivation therapy (ADT) achieves a remission in 80–90% in men with advanced prostate cancer and an average progression-free interval of 12–33 months [4].

Despite the fact that this form of therapy is very successful, it also known for its side effects and the impact of these side effects on the quality of life in addition to the psychological and physical effects influencing the quality of life in the long term. The most common life quality diminishing side effects are reduced libido, depression, fatigue, gynecomastia, hot flushes, obesity, hypertension, insulin resistance, and osteoporosis [5].

Non-pharmaceutical lifestyle interventions aimed to reduce side effects while aiming to leave control in the hands of the patient are very important. They may prevent medicalization and are thought to increase quality of life.

Previous reviews have shown that physical activity may alleviate side effects of ADT [6, 7]. These reviews mainly focused on the effects of different types of exercise, but did not include all types of lifestyle interventions. The aim of this research is to provide a comprehensive overview of recent physical and psychological lifestyle interventions to reduce different side effects of ADT and to investigate the impact of these interventions on quality of life.

## Methods

### Data acquisition and search strategy

A literature search was conducted using the electronic databases Medline and PubMed. The literature search included relevant publications until January 25, 2019. Predefined search terms were used to identify articles concerning interventions to reduce side effects of ADT used as a therapy for prostate cancer.

### Eligibility criteria

To be eligible, the study population had to consist of patients diagnosed with local or advanced PCa in whom ADT was started. Studies had to be randomized controlled trials (RCTs) that focused on side effects of ADT and lifestyle interventions to reduce those side effects. For this review, we selected studies concerning the following psychological effects: quality of life (health-related quality of life and disease-specific quality of life), fatigue, reduced libido, and depression. We selected studies investigating the following physical side effects: gynecomastia, hot flushes, osteoporosis, obesity, or a decreased cardiovascular health. Lifestyle interventions were defined as interventions that included any dietary or behavioral components. Studies reporting on medical therapies to diminish side effects were excluded, as were studies in which the participants stopped their ADT and studies not related to humans.

### Screening of abstracts and full-text articles

A search was performed for abstracts that may be used for inclusion. Abstract screening was done according to predefined inclusion and exclusion criteria. Full-text original articles were retrieved from the selected abstracts; only articles published in English or Dutch that were available for review were selected. Abstracts and original articles were independently assessed by two reviewers for eligibility (MG and NP). Disagreements were solved by consensus procedure in which an independent third author (HBLG) was conducted.

**Fig. 1:**
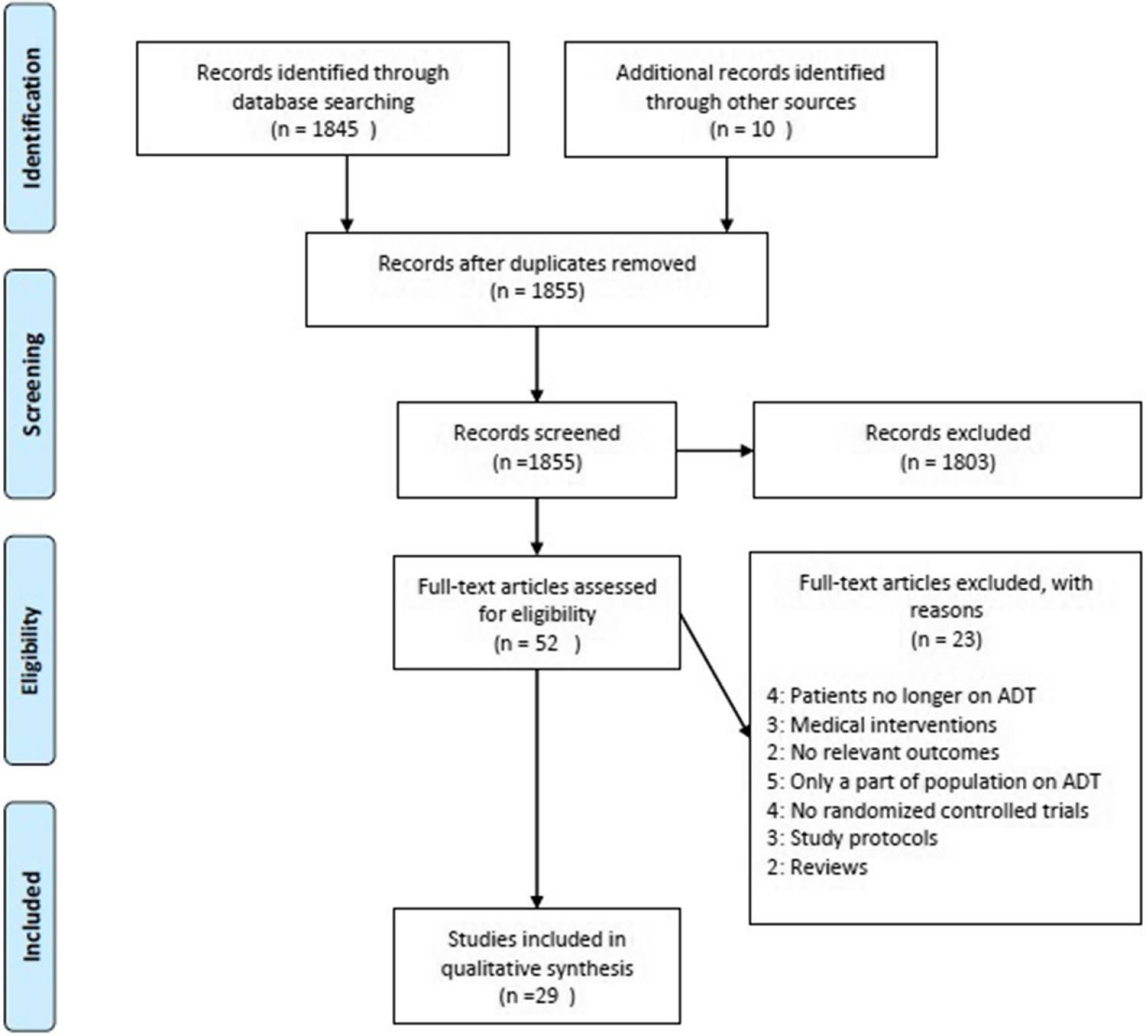
Flow chart of the search and selection process

Subsequently, reference lists of all full-text articles were screened to identify additional relevant articles not found in the PubMed and Medline databases. The final number of included and excluded studies is illustrated in the Preferred Reporting Items for Systematic Reviews and Meta-analyses (Fig. 1).

### Study quality assessment tool

The Cochrane Collaboration’s tool was used to assess the risk of bias in the randomized controlled trial [8]. Two reviewers (MG and NP) assessed the overall quality. If no agreement could be reached, an independent person was involved (HPB).

### Data synthesis

The following data were independently extracted from full-text articles by two reviewers: study year, study design, study population, mean age, type of interventions, duration and frequency of interventions, relevant study outcomes, and methods to assess these outcomes.

## Results

### Search results and analysis

Our literature search identified 1961 of which 29 articles were included in this analysis. The studies were all RCTs investigating the effect of life style interventions to mitigate ADT-induced side effects. Twenty studies investigated exercise modalities, two studies investigated dietary advice, and four studies combined these methods. Three studies investigated behavioral components, existing of cognitive behavioral therapy, educational support programs, or self-education. Study characteristics are depicted in Table 1.

**Table 1:** Study characteristics of the included studies

Study year	Patients (*n*)	Age (mean)	Inclusion criteria	Interventions	Duration intervention (weeks) + frequency (*x*/week)	Relevant outcomes	Methods to assess relevant outcomes
Segal [9]	Int: 82 Con: 73 Total: 155	Int: 68.2 Con: 67.7	Local and advanced PCa receiving ADT ≥ 3 months	Supervised resistance exercise	Duration: 12 Frequency: 3	Fatigue Overweight Quality of life	FACT-F BMI, waist circumference, skinfolds thickness FACT-P
Taylor [10]	Life: 46 Educ: 51 Con: 37 Total: 134	Int + Con: 69.2	PCa treated with ADT	Lifestyle activity program Educational support program	Duration 24 Frequency: week 1–16: 1 4 week: 2	Overweight Depression Quality of life	BMI: Waist and hip circumferences, waist-hip ratio CES-D SF-36, STAI
Sharma [11]	Soy: 20 Con: 19 Total 39	Soy: 69.2 Con: 69.0	PCa treated with ADT	Soy protein: 20 g	Duration: 12 Frequency: 7	Fatigue Libido and sexual function Hot flushes Quality of life	SF-36 IIEF, WSFS Blatt-Kupperman scale SF-36
Culos-Reed [12]	Int: 53 Con: 47 Total: 100	Int: 67.2 Con: 68	PCa localized or metastatic and expect to receive ADT for ≥ 6 months	Supervised and unsupervised aerobic + resistant exercise	Duration: 16 Frequency: 3–5	Fatigue Overweight Cardiovascular Libido Depression Quality of life	FSS BMI, waist-to-hip ratio RR EPIC CES-D EORTC-QLQ-C30, EPIC
Galvao [13]	Int: 29 Con: 28 Total: 57	Int: 69.5 Con: 70.1	Locally or advanced PCa (without bone metastasis) with prior exposure ADT > 2 months	Supervised aerobic + resistance exercise	Duration: 12 Frequency: 2	Fatigue Overweight Cardiovascular Insulin resistance Quality of life	QLQ-C30, SF-36 Dual X-ray absorptiometry: total lean body mass, regional lean mass: upper limb, lower limb, appendicular skeletal muscle total fat mass, trunk fat mass, and percentage body fat, Blood samples: total cholesterol, LDL, HDL, triglycerides, CRP 400 m walk Blood samples: Insulin + glucose EORTC-QLQ-C30; SF-36
Bourke [14]	Int: 25 Con: 25 Total: 50	Int: 71.3 Con: 72.2	Non localized PCa who receiving ADT ≥ 6 months	Supervised + unsupervised aerobic + resistance exercise	Duration: 12 Frequency: Resistance + aerobic: week 1–6: 1; 7–12:2x Eating seminars: 1x	Fatigue Overweight Insulin resistance Quality of life	FACT-F BMI; weight; waist-to-hip ratio Blood biomarkers: insulin; IGF-1 FACT-P; FACT-G
Cormie [15]	Int: 29 Con: 28 Total: 57	Int: 69.5 Con: 70.1	Locally PCa, on ADT ≥ 2 months and remained hypogonadal for ≥ 6 months	Supervised aerobic + resistance exercise	Duration: 12 Frequency: 2	Libido and sexual function Quality of life	QLQ-PR25 SF-36
Hvid [16]	Int: 10 Con: 9 Total: 19	Int: 67.8 Con: 68.5	PCa with ADT > 3 months Control group: healthy males	Supervised endurance Exercise	Duration: 12 Frequency: 3	Overweight Cardiovascular Insulin resistance	Dual x-ray absorptiometry: body lean mass, fat mass, trunk fat mass MR: femoral to liver distance, visceral fat mass, skin fat mass, intermuscular adipose tissue Tissue samples: m. vastus lateralis Blood biomarkers: total cholesterol, LDL, HDL, triglycerides VO2 Max OGTT Euglycemic–hyperinsulinemic clamp Blood samples: glucose, insulin Glucose kinetics calculations
Santa Mina [17]	Aer: 32 Res: 34 Total: 66	Aer: 72.1 Res: 70.6	PCa currently receiving ADT ≥ 12 months	Supervised and unsupervised aerobic or resistance exercise	Duration: 26 Frequency Total: 3–5 Supervised: 1 × 2 week	Fatigue Overweight Gynecomastia Cardiovascular Quality of life	FACT-F BMI, Body fat, waist circumference, body fat: Skinfolds: chest, abdomen + thigh Chest skinfold thickness VO2 max FACT-P; PORPUS
Vitolins [18]	Soy: 30 Ven: 30 V + S: 30 Con: 30 Total:120	Soy: 71 Ven: 67 V + S: 69 Con: 67	Locally advanced or metastatic PCa on ADT and having hot flushes	Soy protein: 20 g Venlafaxine	Duration: 12 Frequency: daily	Hot flashes Quality of life	HFSSS FACT-P; FACT-G
Walker [19]	Int: 20 Con: 20 Total: 40	NR	PCa on ADT	Education booklet on side effects Educational review survey	NA	Libido	PAIR inventory: DAS scale Sexual activity
Bourke [20]	Int: 50 Con: 50 Total: 100	Int: 71 Con: 71	Locally advanced or metastatic PCa, previously on ADT ≥ 6 months and planned long term on ADT	Aerobic + resistance Exercise + dietary advice	Duration: 12 Frequency Resistance: week 1–6: 2x; 7–12: 1x Aerobic: week 1-6: 1; 7–12: 2x Dietary: 1 × 2 weeks	Fatigue Overweight Cardiovascular Quality of life	FACT-F BMI; weight Systolic blood pressure, aerobic exercise tolerance FACT-P
Uth [21]	Int: 29 Con: 28 Total: 57	Int: 67.1 Con: 66.5	Advanced or locally advanced PCa with ADT or surgical castration ≥ 6 months	Supervised football training	Duration: 12 Frequency: week 1–8: 2 week 9–12: 3	Overweight Cardiovascular	Dual X-ray absorptiometry: lean body mass, android, gynoid total body fat mass: BMI; waist circumference; hip circumference VO2 max: 4-min walking test; incremental test to exhaustion Pulmonary gas exchange measurements Heart rate monitors
Winters- stone [22, 23]	Int: 29 Con: 22 Total: 51	Int: 69.9 Con: 70.5	Localized or metastatic PCa currently receiving ADT	Int: Supervised + unsupervised resistance exercise Cont: Stretching	Duration: 52 Frequency: 3	Fatigue Overweight Insulin resistance Quality of life	SCFS BMI Dual X-ray absorptiometry: Fat mass; Bone-free lean mass; trunk fat mass Blood biomarkers: insulin; IGF-1 LLFDI
Cormie [24]	Int: 32 Con: 31 Total: 63	Int: 69.6 Con: 67.1	PCa within first 10 days on ADT and anticipated to remain on ADT ≥ 3 months	Supervised aerobic + resistance exercise	Duration: 12 Frequency: 2	Fatigue Overweight Cardiovascular Osteoporosis Libido Insulin resistance Depression Quality of life	FACIT-fatigue Dual X-ray absorptiometry: body lean mass, fat mass, appendicular lean mass, trunk fat mass, visceral fat mass Blood biomarkers: CRP VO_2_ max: 400-m walk test RR: brachial Dual X-ray absorptiometry: BMD: hip, lumbar spine, whole body Blood biomarkers: alkaline phosphatase, P1NP, N-telopeptide, N-telopeptide/creatinin*e* ratio, vitamin D QLQ-PR25 Blood biomarkers: Insulin, glucose BSI-18 QLQ-PR25; SF-36
Nilsen [25]	Int: 28 Con: 30 Total: 58	Int: 66 Con: 66	Locally advanced PCa receiving RT after neo-adjuvant ADT for 6 months and adjuvant ADT 9–36 months	Supervised resistance exercise	Duration: 16 Frequency: 3	Overweight Fatigue Osteoporosis Cardiovascular Quality of life	Dual x-ray absorptiometry: lean body mass, Regional LBM: (trunk, lower extremities, upper extremities, appendicular skeletal mass), fat mass, fat percentage, body mass, BMI EORTC- QLQ-C30 Dual X-ray absorptiometry: BMD: total, total lumbar spine, hip, trochanter and femoral neck Shuttle walk test EORTC-QLQ-C30
O’neill [26]	Int: 47 Con: 47 Total: 94	Int: 69.7 Con: 69.9	PCa treated ADT for ≥ 6 months and planned to continue for ≥ 6 months or commencing to start ≥ 6 months	Aerobic exercise + dietary advice	Duration: 26 Frequency: Walking: 5	Overweight Fatigue Cardiovascular Quality of life	Weight, BMI, fat mass, lean muscle mass, waist-to-hip ratio, mid-upper arm muscle area Fat mass: Skinfold thickness FSS 400-m walk test FACT-P; PSS
Stefanopoul ou [27]	Int: 33 Con: 35 Tot: 68	Int: 68.0 Con: 68.7	Localized or metastatic PCa undergoing ADT	CBT booklet	Duration: 4	Hot Flushes Depression Quality of life	HFNS HADS EORTC-QLQ-C30; EORTC- QLQ-PR25
Gilbert [28]	Int: 25 Con: 25 Total: 50	Int: 70.1 Con: 70.4	PCa receiving ADT ≥ 6 months + radiotherapy	Supervised + unsupervised aerobic, balance, resistance exercise + Dietary advice: healthy-eating seminars	Duration 12 Frequency: Training: 3 Diet: 1 × 2 week	Overweight Cardiovascular	BMI, weight Blood biomarkers: total cholesterol, LDL, HDL, triglycerides RR: Brachial artery FMD, GTN-arterial dilatation VO2 max: Exercise tolerance: walking test
Nilsen [29]	Int: 16 Con: 15 Total: 31	Int: 66 Con: 65	Locally advanced PCa receiving RT after neo-adjuvant ADT for 6 months and adjuvant ADT 9-36 months	Supervised resistance exercise	Duration: 16 Frequency: 3	Overweight	Muscles biopsies m. Vastus lateralis: protein concentrations, HSP70, Alpha B-crystalline, HSP27, HSP27, HSP60, C OXIV, Citrate synthase Ubiquitin
Nilsen [30]	Int: 12 Con: 11 Total: 23	Int: 67 Con: 64	Locally advanced PCa receiving RT after neo-adjuvant ADT for 6 months and adjuvant ADT 9–36 months	Supervised resistance exercise	Duration: 16 Frequency: 3	Overweight	Muscle biopsies m. Vastus lateralis: histology, muscle fiber CSA, myonuclei, satellite cells, protein concentrations
Sajid [31]	EXCAP: 6 Wii-fit: 8 Con: 5 Total: 19	EXCAP: 75.7 Wii-fit 77.5 Con: 71.8	PCa with ADT, ADT combined with RT	EXCAP: Unsupervised aerobic exercise + resistance exercise Wii-fit: Multi- component exercise on WII	Duration: 6 Frequency: 5	Fatigue Overweight	SPPB Dual X-ray absorptiometry: body fat mass, body lean mass, skeletal muscle mass
Uth [32]	Int: 29 Con: 28 Total: 57	Int: 67.1 Con: 66.5	Locally advanced or metastatic PCa undergoing ADT for ≥ 6 months	Supervised football exercise	Duration: 32 Frequency: week 1–: 2 week 9–12: 3 week 12–32: 2	Overweight Osteoporosis	Dual X-ray absorptiometry: body lean mass, fat mass, percentage fat mass, Dual x-ray absorptiometry: BMD: total hip, femoral, lumbar spine, Blood samples: P1NP, osteocalcin, CTX
Uth [33]	Int: 29 Con: 28 Total: 57	Int: 67.1 Con: 66.5	Locally advanced or metastatic Pca undergoing ADT for ≥ 6 months	Supervised football exercise	Duration: 12 Frequency: wk 1-8: 2 wk 9-12: 3	Osteoporosis	Dual X-ray absorptiometry BMC: total body, legs BMD: total body, legs Blood biomarkers: CTX, P1NP, osteocalcin
Kim [34]	Int: 26 Con: 25 Total: 51	Int: 70.5 Con: 71.0	PCa Stage I to III receiving ADT	Unsupervised strength training + resistance exercise	Duration: 26 Frequency: 3-5	Osteoporosis Quality of life	Dual x-ray absorptiometry: total BMD, regional BMD: Total hip, femur neck, lumbar spine, Blood samples: bs-ALP, Ntx FACT-P
Taaffe [35]	ILRT: 58 ART: 54 Con: 51 Total:163	ILRT:68. 9 ART: 69 Con: 68.4	PCa with ADT exposure ≥ 2 months, anticipated to receive ADT following 12 months	Supervised impact + resistance exercise (ILRT) Supervised + unsupervised aerobic + resistance exercise (ART)	Duration: 52 Frequency: ILRT + AER: 2	Fatigue Cardiovascular	EORTC-QLQ-C30; SF-36 400 m walk
Wall et al. [36]	Int: 50 Con: 47 Total: 97	Int: 69.1 Con: 69.1	Localized PCa on ADT for ≥ 2 months	Supervised and unsupervised aerobic + resistance exercise	Duration: 26 Frequency: 2	Overweight Cardiovascular Insulin resistance	Dual X-ray absorptiometry: lean body mass, fat mass, trunk fat mass, percentage body fat, appendicular fat mass, weight Blood biomarkers: CRP Respiratory gas analysis: fat oxidation, carbohydrate oxidation Blood samples: LDL; HDL; cholesterol, triglycerides VO2Max; RR: brachial Applanation tonometry: arterial stiffness, aortal blood pressure Carotid-to-radial pulse-wave Blood biomarkers: Insulin: Hba1c, glucose
Dawson [37]	Int: 13 Con: 19 Total: 32	Pro: 68.6 Con: 66.3	PCa with ADT	Supervised resistance training	Duration: 12 Frequency: 3	Fatigue Overweight Cardiovascular Insulin resistance Depression Quality of life	BFI Dual X-ray: appendicular skeletal mass, lean body mass, fat mass, percentage body fat, waist circumference Systolic blood pressure, diastolic blood pressure Blood biomarkers: Glucose CES-D FACT-G; FACT-P

*RCTs* randomized controlled trials; *Int* intervention group, *Con* control group, *PCa* prostate cancer, *ADT* androgen deprivation therapy, *RT* radiotherapy, *Ven/V* venlafaxine, *Soy/S* soy protein, physical examinations, *BMI* body mass index, *LBM* lean body mass, *BMD* bone mineral density, *BMC* bone mineral content, *PPB* positive pressure, breathing, *RR* blood pressure, *OGTT* oral glucose tolerance test, *VO2 max* maximal oxygen consumption, *FMD* flow-mediated dilatation, *GTN-arterial dilatation* glyceryl trinitrate-arterial dilatation

*Blood biomarkers* IGF-1: Insulin Growth Factor-1, IGFBP: Insulin-Like Growth Factor-Binding Peptide, IGFBP-1: Insulin-Like Growth Factor-Binding Peptide-1, IGFBP-3: Insulin-Like Growth Factor-Binding Peptide-3, Hba1c: Glycosylated Hemoglobin type A1c, LDL: Low Density Lipoprotein, HDL: High Density Lipoprotein, CRP: C-Reactive Protein, CTX: Cyclophosphamide, P1NP: Procollagen Type 1, bs-ALP: bone-specific Alkaline Phosphatase, NTX: N-Telopeptide of type I collagen

*Questionnaires* CES-D: Center for Epidemiologic Studies Depression scale, HADS: Hospital Anxiety and Depression Scale, DAS scale: Depression, Anxiety and Stress scale, BFI: Big Five Inventory, BSI-18: Brief Symptom Inventory 18, FSS: Fatigue Severity Scale, FACT-P: Functional Assessment of Cancer Therapy-Prostate; FACT-G: Functional Assessment of Cancer Therapy General; FACT-F: Functional Assessment of Cancer Therapy Fatigue POMS: Profile Of Mood Status; EIFI-exhaustion: The Exercise-Induced Feeling Inventory EORTC-QLQ-C30: European Organization for Research and Treatment of Cancer Quality of Life Questionnaire C30, EORTC-QLQ-PR25: European Organization for Research and Treatment of Cancer Quality of Life Questionnaire Prostate-Specific 25-item, SF-36: short-form health survey (36), EPIC: Expanded Prostate Cancer Index, HFSS: Hot Flashes Severity Score, HFNS: Hot Flushes and Night Sweats, HFSSS: Hot Flushes Severity Symptom Score, PAIR inventory: Personal Assessment of Intimacy in Relationships, SPPB: Short Physical Performance Battery, IIEF: International Index of Erectile Function, WSFS: Watts Sexual Functioning Scale; PORPUS: Patient-Oriented Prostate Utility Scale, PSS: Perceived Stress Scale, LLFDI: Late Life Function and Disability Instrument, STAI: State-Trait Anxiety Inventory

Table 2 shows an overview of RCTs testing mitigation strategies for ADT-induced side effects, categorized per side effect. Table 3 summarizes the quality scores of the RCTs. The quality differed between the studies. The most common source of methodological bias was a lack of proper blinding procedures. Almost one-third of the studies presented incomplete outcome data.

**Table 2:** Overview of RCTs testing mitigation strategies for ADT categorized per side effect

Side effect	Intervention	Study	Outcomes	Methods to measure outcome
Quality of life	Aerobic training	Santa Mina et al. [17]	No difference	PRO (FACT-P; PORPUS)
	Aerobic training + dietary advice	O’neill et al. [26]	Improvement in the individual functional well-being subscale (*P* = 0.04) measured with FACT-P at 26 weeks	PRO (FACT-P; PSS)
	Aerobic + Resistance training	Culos-Reed et al. [12]	No difference	PRO (EORTC-QLQ-C30, EPIC)
		Galvao et al. [13]	Significant improvement for general health (*P* = 0.022), vitality (*P* = 0.19) and physical health score (*P* = 0.02) measured with SF-36 Significant improvement in role (*P* < 0.001), cognitive (*P* = 0.007), nausea (*P* = 0.025), dyspnea (*P* = 0.17) measured with QLQ-C30 after 12 weeks	PRO (QLQ-C30; SF-36)
		Bourke et al. [14]	No difference	PRO (FACT-P; FACT-G)
		Cormie et al. [24]	Significant improvement in social functioning (*P* = 0.015), mental health domains (*P* = 0.006) and the mental health composite score (*P* = 0.022) after 12 weeks measured with SF-36	PRO (QLQ-PR25; SF-36)
	Aerobic + resistance + dietary advice	Cormie et al. [15]	Significant difference in perceived general health (*P* = 0.022), vitality (*P* = 0.019); physical health composite (*P* = 0.02) subscales measured with SF-36 at 12 weeks	PRO (SF-36)
		Bourke et al. [20]	Significant improvement after 12 weeks (*P* = 0.001), no change after 24 weeks measured with FACT-P	PRO (FACT-P)
	Cognitive behavioral therapy	Stefanopoulou et al. [27]	No differences	PRO (EORTC-QLQ-C30; EORTC-QLQ-PR25)
	Resistance training	Segal et al. [9]	Increase in QOL, compared with a decrease for the control group (*P* = 0.001) measured with FACT-P after 12 weeks	PRO (FACT-P)
		Taylor et al. [5]	No differences	PRO (SF-36, STAI)
		Santa Mina et al. [17]	No differences	PRO (FACT-P; PORPUS)
		Nilsen et al. [25]	No differences	PRO (EORTC-QLQ-C30)
		Kim et al. [34]	No differences	PRO (FACT-P)
		Dawson et al. [37]	Significant improvement in quality of life measured with FACT-G (*P* = 0.048) and FACT-P (*P *= 0.04)	PRO (FACT-G; FACT-P)
	Soy protein	Sharma et al. [11]	No differences	PRO (SF-36)
		Vitolins et al. [18]	Improvements in emotional (*P* = 0.029) and functional subscales (*P* = 0.041) and in FACT-G (*P* = 0.025) and FACT-P total scores. (*P* = 0.048)	PRO (FACT-P; FACT-G)
Depression	Aerobic + resistance training	Culos-Reed et al. [12]	No differences	PRO (CES-D)
		Santa Mina et al. [17]	No differences	PRO (FACT-F)
		Cormie et al. [24]	No differences	PRO (BSI-18)
	Cognitive behavioral therapy	Stefanopoulou et al. [27]	No differences	PRO (HADS)
	Educational support program	Taylor et al. [10]	No differences	PRO (CES-D)
	Lifestyle activity program	Taylor et al. [10]	No differences	PRO (CES-D)
	Resistance training	Dawson et al. [37]	No differences	PRO (CES-D)
Fatigue	Aerobic training	Santa Mina et al. [17]	No differences	PRO (FACT-F)
	Aerobic training + dietary advice	O’neill et al. [26]	No differences	PRO (FSS)
	Aerobic + resistance training	Cormie et al. [24]	Significant reduction (*P* = 0.042) measured with FACIT-fatigue	PRO (FACIT-fatigue)
		Culos-Reed et al. [12]	No difference	PRO (FSS)
		Galvao et al. [13]	Significant reduction in fatigue (*P* = 0.021) and increased vitality (*P* = 0.019) measured with SF-36 and QLQ-C30 resp.	PRO (QLQ-C30, SF-36)
		Santa Mina et al. [17]	No differences	PRO (FACT-F)
		Sajid et al. [31]	EXCAP: Significant reduction (*P* = 0.04) measured with SPPB Wii-fit: no differences	PRO (SPPB)
		Taaffe et al. [35]	Significant reduction in fatigue (*P* = 0.005) and increased vitality (*P* < 0.001) measured with EORTC-QLQ-C30 and SF-36 resp.	PRO (EORTC-QLQ-C30; SF-36)
	Aerobic + resistance training + dietary advice	Bourke et al. [14]	Significant reduction at 12 weeks (*P *= 0.002) and 6 months (*P* = 0.006) measured with FACT-F	PRO (FACT-F)
		Bourke et al. [20]	Significant reduction at 12 weeks (*P* < 0.001) and 6 months (*P* = 0.010) measured with FACT-P	PRO (FACT-P)
	Impact + resistance training	Taaffe et al. [35]	Significant reduction of fatigue (*P* = 0.005) and increased vitality (*P* < 0.001)	PRO (EORTC-QLQ-C30; SF-36)
	Resistance training	Segal et al. [9]	Significant reduction (*P* = 0.002)	PRO (FACT-F)
		Sharma et al. [11]	Significant reduction after 12 weeks (*P* = 0.010) and 24 weeks (*P* = 0.002) Per-group analysis: significant reduction after 24 weeks (*P* = 0.04)	PRO (SF-36)
		Santa Mina et al. [17]	No differences	PRO (FACT-F)
		Nilsen et al. [25]	No differences	PRO (EORTC- QLQ-C30)
		Dawson et al. [37]	No differences	PRO (BFI)
	Soy protein	Sharma et al. [11]	No differences	PRO (SF-36)
Decreased libido + sexual function	Aerobic + resistance training	Culos-Reed et al. [12]	No difference between groups or effect over time in sexual function	PRO (EPIC)
		Cormie et al. [15]	Significant difference in percentage of major interest in sex (*P* = 0.024) and maintenance in level of sexual activity (*P* = 0.045) No change in any level of sexual interest or sexual function	PRO (QLQ-PR25)
		Cormie et al. [24]	Significant less decline in sexual function (*P* = 0.028), no difference in sexual activity Per-group-analysis over time: decline in sexual activity (*P* = 0.012), no change in sexual function	PRO (QLQ-PR25)
	Information booklet	Walker et al. [19]	No significant differences. Sexual activity at baseline: 64.3% versus 38.5% (control), after 6 months: 25% versus 0% (control)	PRO (PAIR inventory: DAS scale, sexual activity)
	Soy protein	Sharma et al. [11]	No differences	PRO (IIEF, WSFS)
Overweight	Aerobic training	Hvid et al. [16]	Body composition: no differences Per-group analysis: reduction in BMI (*P* < 0.0001), weight (*P* < 0.0001), fat mass (*P* < 0.01), fat percentage (*P* < 0.05), trunk fat mass (*P* < 0.01) Blood biomarkers: no differences Per-group analysis: Increase in HDL over time. No differences in LDL, total cholesterol, triglycerides	Body composition and blood biomarkers
		Santa Mina et al. [17]	No differences	Body composition
	Aerobic training + dietary advice	O’neill et al. [26]	Body composition: significant reduction (all *P* = 0.001), waist-to-hip ratio (*P* = 0.009) No difference in lean body mass or mid-upper arm muscle area	Body composition
	Aerobic + resistance training	Culos-Reed et al. [12]	Body composition: significant reduction in waist girth (*P* = 0.044) and neck girth (P-0.019), no difference in BMI Per-group analysis: no difference in BMI	Body composition
		Galvao et al. [13]	Body composition: significant increase in total lean mass (*P* = 0.047), upper limb (*P* < 0.001), lower limb (*P* = 0.019), appendicular skeletal mass (*P* = 0.03). No change in weight total body fat mass trunk fat mass or percentage body fat Blood biomarkers: Significant decrease in CRP (*P* = 0.008). No change in other markers	Body composition and blood biomarkers
		Cormie et al. [24]	Body composition: significant reduction in appendicular lean mass (*P* = 0.019), whole body fat mass (*P* = 0.001), whole body percentage fat (*P* < 0.001), trunk fat mass (*P* = 0.008). No differences in total body mass, visceral fat mass or body lean mass Blood biomarkers: significant reduction in HDL total cholesterol ratio (*P* = 0.028). No differences in other biomarkers	Body composition and blood biomarkers
		Sajid et al. [31]	EXCAP: Body composition: no differences Wii-fit: Body composition: decrease in lean mass for the Wii-fit group (*P* = 0.045),	Body composition
		Wall et al. [36]	Body composition: increase in lean body mass (*P* = 0.015), decrease in total fat mass (*P* = 0.020), trunk fat mass (*P* < 0.001), body fat percentage (*P *= 0.001), no change in weight Blood biomarkers: no differences	Body composition and blood biomarkers
	Aerobic + resistance training + dietary advice	Bourke et al. [14]	No differences	Body composition
		Bourke et al. [20]	No differences	Body composition
		Gilbert et al. [28]	Body composition: significant increase in skeletal muscle mass (*P* = 0.03), no difference in BMI or body fat mass Blood biomarkers: no differences	Body composition and blood biomarkers
	Educational support program	Taylor et al. [10]	No differences	Body composition
	Football training	Uth et al. [21]	Significant increase in lean body mass (*P* = 0.02), no change in body fat percentage, body fat mass Per-group analysis: significant increase in lean body mass (*P* = 0.02), no change in body fat percentage or body fat mass	Body composition
		Uth et al. [32, 33]	No differences	Body composition
	Resistance exercise	Uth et al. [32, 33]	No differences	Body composition
		Segal et al. [9]	No differences	Body composition
		Santa Mina et al. [17]	No differences	Body composition
		Winters-stone [23]	Significant reduction in total fat mass in covariance analysis (*P* = 0.02), not in ITT analysis No significant change in body lean mass, percentage body fat, weight or trunk fat mass	Body composition
		Nilsen et al. [25]	Significant increase in LBM in lower extremities (*P* = 0.002) and upper extremities (*P* = 0.048), appendicular skeletal muscle mass (*P* = 0.001). No change in total, trunk lean mass, total fat mass, trunk fat mass, percentage fat mass, weight, or BMI	Body composition
		Nilsen et al. [29, 30]	No significant difference in mitochondrial proteins or indicators of muscle cellular stress: HSP70 B-crystallin; ubiquitin; ubiquittinated proteins Per-group analysis: change over time in HSP70 (*P* = 0.03)	Muscles biopsies m. Vastus lateralis: protein concentrations, HSP70, Alpha B-crystalline, HSP27, HSP27, HSP60, COXIV, Citrate synthase Ubiquitin
		Nilsen et al. [29, 30]	Significant change in total muscle fiber in favor for intervention group (*P* = 0.04) Significant increase in type II fibers for intervention group (*P* = 0.03); no change myonuclei number	Muscle biopsies m. Vastus lateralis: histology, muscle fiber CSA, myonuclei, satelite cells, protein concentrations
		Dawson et al. [37]	Significant increase in lean mass, appendicular skelet mass (*P* = 0.02), sacropenic index (*P* = 0.02), fat free mass (*P* = 0.04). Significant decrease in waist circumference (*P *= 0.01), and percentage body fat (*P* = 0.01). No difference in fat mass or total mass Blood biomarkers: No difference	Body composition
Hot flushes	Cognitive Behavioral Therapy	Stefanopoulou et al. [27]	Decreased hot flashes problem rating (*P* = 0.001) and reduced frequency at 6 weeks (*P* = 0.02). No difference at 32 weeks	PRO (HFNS)
	Soy protein	Sharma et al. [11]	Difference in favor of the placebo group at 12 weeks (*P* = 0.04). No difference at 6 weeks Per-group analysis: no difference in vasomotor symptoms	PRO (Blatt-Kupperman scale)
		Vitolin et al. [18]	No differences Per-group analysis: significant decrease of vasomotor symptoms (*P* < 0.001), hot flushes severity (*P* < 0.001), hot flushes symptom severity score (*P* < 0.001)	PRO (HFSS, HFSSS)
Gynaecomastia	Aerobic training	Santa Mina et al. [17]	No difference	Body composition
	Resistance training	Santa Mina et al. [17]	No difference	Body composition
Cardiovascular	Aerobic training	Hvid et al. [16]	No significant difference in VO_2_ max (ml(O_2_)/min per kg), VO2max (ml(O_2_)/min Per-group analysis: increase in VO2 max (ml(O_2_)/min per kg) (*P* < 0.0001), VO_2_ max (ml(O_2_)/min) (P < 0.001).	Physical test (VO_2_max)
			No difference	
		Santa Mina et al. [17]	No difference	Physical test (VO_2_max)
	Aerobic training + dietary advice	O’neill et al. [26]	Significant change (P = 0.001)	Physical test (6-m walk test)
	Aerobic + resistance training	Culos-Reed et al. [12]	No difference Per-group analysis: significant reduction of systolic blood pressure (P = 0.011) and diastolic blood pressure (P = 0.004)	Blood pressure
		Galvao et al. [13]	No difference	Physical test (400 m walk test)
		Cormie et al. [24]	Significant increase in VO2 max (*P* = 0.004) and 400 m walk (*P* = 0.009) No change in blood pressure	Physcial test (VO_2_ max: 400-m walk test) Blood pressure
		Taaffe et al. [35]	No differences Per-group analysis: cardiovascular fitness improved ART: *P* < 0.001	Physical test (400 m walk)
		Wall et al. [36]	Significant increase in VO2 Max (L min) and VO2 Max (ml/kg) (*P* = 0.033), fat oxidation (*P *= 0.037) No change in RMR, carbohydrate oxidation, peripheral systolic, diastolic RR or MAP, central diastolic RR or MAP, peripheral augmentation index, central augmentation pressure, central augmentation, index, pulse-wave velocity	Physical test (Respiratory gas analysis, VO2 Max), blood pressure, blood samples
	Aerobic + resistance training + dietary advice	Bourke et al. [20]	Significant improvement in aerobic exercise tolerance at 12 weeks (*P* < 0.001) and 6 months (*P* < 0.001) No difference in systolic blood pressure	Physcial test (aerobic exercise tolerance), blood pressure
	Aerobic, balance, resistance exercise + dietary advice	Gilbert et al. [28]	Treadmill walk time improved at 12 and 24 weeks (*P* < 0.001) Difference in FMD at 12 weeks (*P* = 0.04). No difference in GTN dilatation, systolic blood pressure or diastolic blood pressure	Physical test (VO2 max, exercise tolerance: walking test), blood pressure
	Football training	Uth et al. [21]	No significant difference between groups Per-group -analysis: VO2 max increased in the intervention group (1.0 ml/kg/min) *P* = 0.02	Physical test (VO2 max: 4-min walking test; incremental test to exhaustion, pulmonary gas exchange measurements; heart rate monitors)
	Impact + resistance training	Taaffe et al. [35]	No differences	Physical test (400 m walk)
	Resistance training		VO2max improvement (*P* = 0.41), also after adjustment for covariates (*P* = 0.38)	
		Santa Mina et al. [17]	Significant improvement compared to control group	Physical test (VO2 max)
		Dawson et al. [37]	No differences	Blood pressure
**Insulin resistance**	Aerobic training	Hvid et al. [16]	No difference in fasting glucose, glucose AUC or insulin AUC compared to control group Per-group analysis: significant change in fasting glucose (*P* < 0.05), no change in glucose AUC or insulin AUC	Physical test (VO2 Max OGTT Euglycemic– hyperinsulinemic clamp), blood biomarkers
	Aerobic + resistance training	Galvao et al. [13]	No differences	Blood biomarkers
		Cormie et al. [24]	No differences	Blood biomarkers
		Wall et al. [36]	Significant change for glucose (*P* < 0.001). No change for Hba1c or insulin	Blood biomarkers
	Aerobic + resistance training + dietary advice	Bourke et al. [14]	No differences	Blood biomarkers
	Resistance training	Winters-stone [23]	No differences	Blood biomarkers
		Dawson et al. [37]	No differences	Blood biomarkers
**Osteoporosis**	Aerobic + resistance training	Cormie et al. [24]	No differences	Body composition, blood biomarkers
	Football training	Uth et al. [32]	Bone mineral content: significant increment in total body BMC (*P* = 0.013), leg BMC (*P* < 0.001) Bone mineral density: no differences Blood biomarkers: significant change P1NP (*P* = 0.008) and osteocalcin (*P* = 0.002), no change in CTX	Body composition, blood biomarkers
		Uth et al. [32]	Bone mineral density: significant increment in left total hip (*P* = 0.030), right total hip (*P* = 0.015), left femoral shaft (*P* = 0.015), right femoral shaft (*P* = 0.016). No difference in right femoral neck, left femoral neck, lumbar spine L2–L4 Blood biomarkers: no differences	Body composition, blood biomarkers
	Resistance training	Nilsen et al. [25]	No differences	Body composition
		Kim et al. [34]	No differences	Body composition

**Table 3:** Methodological quality assessment tool for randomized controlled trials

		Random sequence generation (Selection bias)	Allocation concealment (Selection bias)	Blinding of participants and personnel (performance bias)	Blinding of outcome assessment (attrition bias)	Incomplete outcome data (attrition bias)	Selective reporting (reporting bias)	Other bias
Segal [9]	2003	+	+	−	?	−	+	+
Taylor [10]	2006	−	−	−	+	+	+	−
Sharma [11]	2009	+	+	+	+	?	+	−
Culos-Reed [12]	2010	+	+	−	+	?	+	−
Galvao [13]	2010	+	+	−	−	+	+	+
Bourke [14]	2011	+	?	−	+	+	?	?
Cormie [15]	2013	+	+	−	?	+	+	+
Hvid [16]	2013	−	−	−	+	−	+	−
Santa Mina [17]	2013	?	+	−	+	+	+	−
Vitolins [18]	2013	+	+	+	+	−	−	−
Walker [19]	2013	?	?	−	?	+	+	−
Bourke [20]	2014	+	+	−	−	+	+	+
Uth [21]	2014	+	+	−	+	+	+	−
Winters-Stone [22, 23]	2015	?	?	−	+	+	+	−
Cormie [24]	2015	+	+	−	?	+	+	+
Nilsen [25]	2015	+	?	−	+	+	+	+
O’Neill [26]	2015	+	+	−	−	+	+	?
Stefanopoulou [27]	2015	+	+	−	+	−	+	−
Gilbert [28]	2016	+	+	−	+	+	+	+
Nilsen [29]	2016	+	?	−	+	−	?	−
Nilsen [30]	2016	+	?	−	+	−	?	−
Sajid [31]	2016	+	+	−	+	−	+	+
Uth [32]	2016	+	+	−	+	+	?	−
Uth [33]	2016	+	+	−	+	−	+	−
Kim [34]	2017	+	+	−	−	−	+	−
Taaffe [35]	2017	+	+	−	+	−	+	?
Wall Dawson [36, 37]	2017 2018	+ +	+ +	− −	+ −	− +	+ ?	+ +

Risk of bias summary for each trial included in the systematic review as evaluated by authors

+ low risk of bias; ? Some concerns are risk of bias; − high risk of bias

#### Psychological side effects

##### Quality of life

Health-related quality of life (HRQOL) is perceived physical and mental health over time either by an individual or by a group. The influence of lifestyle interventions on the quality of life for patients on ADT was examined in 16 studies [9–15, 17, 18, 20, 23–27, 34, 36].

Ten different questionnaires were used to measure HRQOL. Some of the questionnaires are developed to examine health-related quality of life in general (STAI; SF-36; EORTC-QLQ- C30; QLQ-PR25; PSS; FACT-G; LFDI). Others are used to investigate prostatic disease-specific quality of life or aspects of quality of life (FACT-P; PORPUS; EPIC). Eleven studies investigated the effects of physical exercises, three combined physical exercises with dietary advice, and two investigated the effect of soy consumption. In four studies, a positive effect of exercise only was noted [9, 13, 24, 36]. Two out of six studies investigating the effect of resistance training found a positive effect on the health-related quality of life and in one of these studies, an improved disease-specific quality of life had been found [36]. Aerobic and resistance training combined showed a positive effect in two out of four studies [13, 24]. In these two studies, only specific domains were significantly improved. For details, see Table 2. Variable results were found when combining exercises with dietary advice. All three studies found positive effects on different aspects of quality of life [15, 20, 26]. Bourke et al. found an improvement in the prostatic disease-specific quality of life after 12 weeks of training, but this was only temporary since the effect disappeared after 24 weeks [20]. One study investigated the effect of cognitive behavioral therapy on quality of life, but failed to show an improvement. Two studies investigated the effect of soy consumption. Vitolins et al. found an improved HRQOL as well as an improved disease-specific quality of life after consumption of soy protein, while Sharma et al. failed to show any effect [11, 18].

##### Depression

In the treatment of PCa, ADT use is associated with depression. Five studies focused on depression [10, 12, 24, 27, 36]. Aerobic and/or resistance training failed to show a beneficial effect [12, 24, 36]. Applied cognitive behavioral therapy (CBT) for a period of four weeks did not show a beneficial effect [27]. A lifestyle activity program or an educational support program did not influence depression scores using CES-D [10].

##### Fatigue

Fatigue is a phenomenon, which is difficult to measure or define. Fatigue is experienced by patients receiving ADT and is associated with decreased levels of testosterone and reduction of skeletal muscle mass may contribute to fatigue. Influence of lifestyle interventions was examined in fourteen studies: ten investigated the effects of physical exercises, three combined physical exercises with dietary advice, and one investigated the effect of soy consumption [9, 11–14, 17, 20, 24–26, 31, 35, 36]. Soy consumption showed no effect [11].

Different exercise modalities yield conflicting results in relation to fatigue. Generally speaking, in half of the studies, a reduction in fatigue was noted. Combining exercises with dietary advice showed a beneficial effect in two studies [14, 20]. Another study failed to show improvement [26].

##### Decreased libido and sexual function

Since androgens play an essential role in maintaining sexuality, e.g., libido and erectile function, it is obvious that ADT causes a decrease in sexual activity and may result in a variety of sexual problems. Five studies investigated methods to counteract decreased libido and erectile function [11, 12, 15, 19, 24]. Aerobic and resistance exercises were examined in three studies in which contradictory findings were reported. In 2013, Cormie et al. found maintenance of major interest in sex as well as sexual activity in the exercise group. No change in sexual function was noted. In 2015, Cormie et al. reported a diminished decline in sexual function in favor of the exercise group. In per-group analysis, a decline in sexual activity was found without change in sexual function [24]. Culos-Reed et al. found no differences [12].

Patient information concerning the side effects of ADT followed by an educational partner session failed to show an effect on libido, measured by the PAIR and DAS-scores which, respectively, assess the current level of intimacy in one’s relationship and the relationship adjustment. Couples participating in the intervention were more successful at maintaining sexual activity [19]. Soy protein consumption did not influence libido or sexual function [11].

#### Physical side effects

##### Gynecomastia

Gynecomastia may develop in ADT. In one study, gynecomastia was measured by skinfold thickness and was not influenced by aerobic or resistance training [17].

##### Hot flushes

Hot flushes are defined as intense heat sensation, flushing, and perspiration involving face and trunk. Anxiety and palpitations may occur. ADT may induce these complaints because the decline in LH and FSH causes the release of hypothalamic catecholamines disrupting the thermoregulation center in the upper hypothalamus. CBT temporarily lowers the occurrence of hot flushes and problem rating in men [27]. Only at 6 weeks, significance was reached, and it was no longer significant at 32 weeks. Two studies examined the effect of soy consumption on hot flushes. One study found a significant decrease of ADT-associated vasomotor symptoms in the soy protein and placebo group [18]. Sharma et al. observed no change.

Surprisingly, a significant difference in favor of the placebo group was found [11].

##### Overweight

ADT may cause metabolic effects including dyslipidemia, elevated fasting serum glucose, weight gain, and increase in fat mass. C-reactive protein (CRP) might be a marker of adverse metabolic effects. Seventeen studies reporting on exercise programs of varying duration, frequency, intensity, and degree of supervision showed conflicting results [9, 10, 12–14, 16, 17, 20–26, 28–32, 36, 37]. These articles mention aspects of body composition amounting to sixteen different items: weight, BMI, waist circumference, hip circumference, waist and neck girth, waist-to-hip ratio, mid-upper arm muscle area, total fat mass, percentage body fat, trunk fat mass, visceral fat mass, body lean mass, appendicular lean mass, and skeletal muscle mass. The effect of aerobic and/or resistance training on body composition was examined in thirteen studies. Three of these studies added dietary advice [14, 20, 28].

Beneficial effects on one or multiple items reflecting body composition were found in eight of these studies (Table 2).

O’neill et al. investigated the effect of aerobic training and dietary advice and showed an improvement in body composition [26]. Two studies examined the effect of supervised football training [21, 32]. One study lasted 12 weeks, the other 32 weeks. Initially, a significant increase in lean body mass was reached after 12 weeks [21]. At 32 weeks, this effect ceased to be significant. Endurance training showed an improvement of body composition [16]. Metabolic syndrome is a clustering of at least three out of five following medical conditions: hypertension, hyperglycemia, abdominal obesity, high serum triglycerides, and a low high-density lipoproteins (HDL).

Blood biomarkers reflecting these changes were examined in six studies. A decrease in HDL/total cholesterol ratio was found by Cormie et al., who investigated the effects of a 12-week program combining aerobic and resistance training [24]. An improvement in HDL was found over time after a program of endurance training [16].

No other studies reported beneficial effects. The influence of aerobic and resistance training on CRP showed contradictory results. One study found a positive effect [13], and two studies found no change [24, 36]. On microscopic level, the effect of resistance training in biopsies of the m. vastus lateralis was reported [29, 30]. An increase in total muscle fibers and type II fibers was shown [29]. Muscle atrophy is associated with reduced mitochondrial function and increased muscle cellular stress, reflected by different heat shock proteins. Only HSP70 improved significantly [30].

##### Cardiovascular health

Factors influencing cardiovascular health are manifold: lipid profile, blood pressure, BMI, endothelial cell function, pro-inflammatory factors, and insulin resistance. ADT has a negative impact on these factors. Besides, there is an association between ADT and the occurrence of serious cardiac arrhythmias. Eleven studies investigated the effect of physical exercise of which three studies added dietary advice [12, 13, 16–18, 24, 26, 28, 36, 37]. The results of these studies are contradictory. With respect to blood pressure, four studies found no effect on systolic or diastolic blood pressure between the groups [12, 20, 24, 28]. Culos-Reed et al. found a change in systolic and diastolic blood pressure over time in the aerobic and resistance training group [12]. Using flow-mediated dilatation (FMD) as a measure, an improvement in the exercise combined with dietary advice group was found [28]. The effect of training on maximum oxygen utilization (VO2 max) was investigated in five studies [17, 18, 24, 36, 37]. Most of these studies consisted of a mixture of aerobic and/or resistance training. Uth et al. used football training. Almost consistently, an improvement was found.

Resistance training scored better in increasing cardiovascular fitness  than aerobic [17, 18]. Four studies found a significant improvement on cardiovascular fitness after physical exercises, measured by different tests [13, 26, 29, 35].

##### Insulin resistance

It is not completely understood how ADT therapy deregulates glucose metabolism. Possibly hypogonadism, secondary to obesity, alters fatty acid metabolism or changes in skeletal muscle may play a role. The influence of exercise on insulin resistance was measured in seven studies [13, 14, 16, 22–24, 36, 37]. One study added dietary advice. A glucose-lowering effect of exercise was shown in two studies [16, 36]. Wall et al. showed an improvement for glucose metabolism in combined aerobic and resistance training [36]. Hvid et al. investigated the effect of endurance training on insulin resistance in men on ADT compared to healthy males and found a difference in fasting glucose over time in the per-group analysis [16].

Using the oral glucose tolerance test (OGTT), no differences over time were found between the groups with regard to fasting glucose, fasting insulin, glucose AUC, and insulin AUC [16]. Biomarkers: IGF-1, insulin, IFGBP-1 and IGFBP-3, and HBA1c failed to show a difference in any of the studies.

##### Osteoporosis

Osteoporosis is a major concern in men undergoing ADT therapy, especially with prolonged use. Five studies investigated the effect of exercise modalities on osteoporosis [24, 25, 32–34]. We were unable to find any studies focusing on dietary advice. Dual x-ray absorptiometry (DEXA-scan) was used in all studies in order to examine bone mineral density (BMD) and bone mineral content (BMC). Blood biomarkers were examined in four studies. Biomarkers were alkaline phosphatase, P1NP, N-telopeptide, N-telopeptide/creatinine ratio, vitamin D, osteocalcin, CTX, NTX, and BS-ALP. Only Uth was able to show a significant improvement in different markers of osteoporosis. He found an increment in BMC after 12 weeks of supervised football training [33]. Initially, BMD remained unchanged after 12 weeks of training [33], whereas after 32 weeks, an increase in BMD was found in the total hip and femoral shaft [32]. P1NP and osteocalcin were the only bone formation markers that showed a significant change. After 12 weeks, there was an increase in these markers [32] After prolonged training (32 weeks), this increase no longer was significant [32].

## Discussion

This study focuses on lifestyle interventions to reduce the deleterious side effects of ADT. Twenty-nine RCTs reported the effect of different lifestyle interventions. The effect of exercises in different modalities and intensities was studied including measures such as dietary advice, self-education, CBT, or educational support program. We excluded therapies like prescribing calcium and vitamin D.

Regarding psychological effects, contradictory results were found. Many different questionnaires were used to examine the effect of lifestyle interventions on HRQOL, which make it hard to compare different outcomes and therefore it is difficult to make a straightforward conclusion about the effect of certain interventions.

Combining exercise with diet seems to have the most beneficial effects on HRQOL; all studies regarding this combination showed a significant improvement [15, 20, 26]. Soy protein consumption was found in only one study to have a beneficial effect.

An earlier study found that ADT use conferred a 41% increased risk of depression [38]. In this review, we were unable to find any difference in the occurrence of depression after applying lifestyle interventions, whereas in two studies an improvement of QOL was noted in absence of improvement of depression score [24, 37]. Tentative explanations for these outcomes can be the following. Both studies used different questionnaires to score quality of life and the occurrence of depression, making it difficult to compare them. In our review, we only noted the truly significant findings, but in the study of Cormie, where an improvement in various subscales of QOL was observed, a borderline significance was found in the depression score with a *P* value of 0054; this might be interpreted as a trend in improvement of physiological well-being [24].

There were many different tools used to examine fatigue. Some used general questionnaires, and others applied prostate-specific tools (FACT-F) [9, 14, 17, 20, 24]. Although there was a lack in using a common tool, different lifestyle interventions reduced fatigue and increased vitality. All studies used in this review examined physical exercises, sometimes combined with dietary advice. However, no attention was given to other lifestyle adjustments, such as smoking, alcohol use, or a balanced diet.

With regard to libido and sexual function, a possible beneficial effect of aerobic and resistance training was found [15, 24]. It is noteworthy that consuming soy protein did not have any effect [11]. Considering the age group for which ADT therapy is applied, we can expect a general natural decline of libido and sexual function.

Gynecomastia may very well occur after weight gain. Only one author reported on this subject [17]. This might reflect a disbelief in the effect of lifestyle changes on gynecomastia. A variety of medical therapies exist such as radiation therapy or lipolysis [39].

Two strategies were examined to reduce vasomotor symptoms. Although soy proteins appears to be successful in treating hot flushes in postmenopausal women [40], in men on ADT, they are unsuccessful. CBT appears to be helpful in managing hot flushes. Limitations of this study were the small sample size and short period of self-guided therapy. Further research might look into additional variables such as caffeine intake and other specific health problems.

In this study, we divided the metabolic changes into three subcategories: overweight, insulin resistance, and cardiovascular health. These categories are interrelated. Concerning insulin resistance, studies failed to show a difference between men with ADT undergoing physical exercises or not, when measured by fasting glucose. Endurance training, however, showed no differences in OGTT between men on ADT undergoing exercises versus healthy men [16]. No trial used the novo diabetes mellitus as an outcome.

Overweight was examined in 17 studies. All studies investigated the effect of exercise, and four studies added nutritional advice. Evidence was found that solely exercise or exercise combined with nutritional advice may decrease overweight. The influence of a dietary advice only on obesity hasn’t been investigated. Earlier studies showed the major impact of an appropriate diet in the prevention of obesity in the healthy old male population [41].

Considering osteoporosis, one study suggests that regularly practicing football may mitigate ADT-induced decline in BMD. However, aerobic or resistance training failed to show this decline. Future studies should investigate the effects of different exercise modalities.

National osteoporosis foundation recommends a daily calcium intake of 1200 mg and supplemental vitamin D of 800–1000 IU to maintain bone health [42]. In this review, there was not one study, which focused on an adequate intake as described above. As mentioned earlier, we did not include RCTs in which calcium and Vitamin D were prescribed. A systematic review conducted in 2012, which investigated the effect of calcium and vitamin D suppletion in men with PCa using ADT showed conflicting effects [43]. It would be interesting to conduct studies in which dietary advice is given to promote sufficient intake of calcium and vitamin D and investigate this effect on the development of osteoporosis in patients on ADT.

A number of limitations in this review must be considered. Firstly, we focused on two databases, PubMed and Medline, since these two are the most widely used and recognized. Secondly, there was substantial heterogeneity in life style interventions, in which some interventions were investigated frequently, while other interventions only once. This makes it impossible to compare the effects of different strategies and draw conclusions about the efficacy of these interventions. Additionally, we did not investigate the effect of duration of exercise, dietary advice, or other behavioral components.

There was a variety in quality of the different RCTs. Especially, the prevention of performance bias was often inadequate, due to the fact it was impossible to blind participants following lifestyle programs. Despite these limitations, evidence suggests that lifestyle interventions may have a beneficial effect on ADT-mediated side effects.

## Conclusions

Adverse effects of ADT are manifold as well as the various lifestyle interventions aimed to alleviate these side effects. Physical exercises demonstrated to have a mainly positive effect on cardiovascular health. Contradictory results were found regarding quality of life, libido, fatigue, insulin resistance, overweight, and osteoporosis. No effect was found regarding depression and gynecomastia. Regarding dietary advice, soy protein showed no beneficial effect on libido or fatigue. No other studies allowed conclusions on dietary advice solely. A positive effect was shown with CBT on the occurrence of hot flushes. Other behavioral components failed to show a significant effect regarding side effects. Further research is necessary to identify the most effective interventions for the individual patient.
